# Atmospheric Turbulence Aberration Correction Based on Deep Learning Wavefront Sensing

**DOI:** 10.3390/s23229159

**Published:** 2023-11-14

**Authors:** Jiang You, Jingliang Gu, Yinglei Du, Min Wan, Chuanlin Xie, Zhenjiao Xiang

**Affiliations:** 1 Institute of Applied Electronics, China Academy of Engineering Physics, Mianyang 621900, China; youjiang09@163.com (J.Y.); gavin51728@163.com (J.G.); boyduyinglei@163.com (Y.D.); wanmin@caep.cn (M.W.); x_cling@163.com (C.X.); 2 Graduate School of China Academy of Engineering Physics, Beijing 100088, China

**Keywords:** adaptive optics (AO), deep learning wavefront sensing (DLWS), aberration correction experiment, CNN, attention mechanism

## Abstract

In this paper, research was conducted on Deep Learning Wavefront Sensing (DLWS) neural networks using simulated atmospheric turbulence datasets, and a novel DLWS was proposed based on attention mechanisms and Convolutional Neural Networks (CNNs). The study encompassed both indoor experiments and kilometer-range laser transmission experiments employing DLWS. In terms of indoor experiments, data were collected and training was performed on the platform built by us. Subsequent comparative experiments with the Shack-Hartmann Wavefront Sensing (SHWS) method revealed that our DLWS model achieved accuracy on par with SHWS. For the kilometer-scale experiments, we directly applied the DLWS model obtained from the indoor platform, eliminating the need for new data collection or additional training. The DLWS predicts the wavefront from the beacon light PSF in real time and then uses it for aberration correction of the emitted laser. The results demonstrate a substantial improvement in the average peak intensity of the light spot at the target position after closed-loop correction, with a remarkable increase of 5.35 times compared to the open-loop configuration.

## 1. Introduction

Adaptive Optics (AO) is a technology that enhances the performance of optical systems by mitigating the impact of dynamic wavefront errors on image quality [1]. It finds wide applications in fields such as astronomical observations and laser communications, and is considered a promising technique for compensating for wavefront distortion caused by atmospheric turbulence or other factors during the imaging process. Wavefront sensing plays a crucial role in this technology, and various wavefront sensors, including Shack-Hartmann Wavefront Sensors (SHWS) [2] and shearing interferometers [3], have been developed to detect wavefront aberrations. However, the measurement accuracy of these sensors can be affected by factors such as weak received beacon light, non-uniform light intensity distribution, or random disturbances during transmission, such as atmospheric turbulence [4,5].

Deep learning has demonstrated powerful capabilities in various fields, including Computer Vision (CV), Natural Language Processing (NLP), and Automatic Speech Recognition (ASR) [6,7]. Its success can be attributed to its ability to learn from large-scale data and extract complex feature representations. As early as 1990, in [8], artificial neural networks were first applied to wavefront aberration identification, constructing a neural network model that maps focal and defocused intensity images to control signals. Subsequently, researchers proposed using three-layer networks to predict intensity images to 4–11 orders of Zernike coefficients [9]. This mapping of focal and defocused intensity images to Zernike coefficients has become a standard paradigm in Deep Learning Wavefront Sensors (DLWS). With the development of deep learning technology, increasingly deeper neural networks are being applied in this field of research.

The emergence of Convolutional Neural Networks (CNNs) has greatly simplified image feature extraction. Through the introduction of innovative structures [10,11,12], CNNs have shown significant improvements in performance. Studies have demonstrated the effectiveness of CNNs in recovering phase information from single-frame images [13]. A mainstream research approach is the establishment of CNN models for predicting Zernike coefficients from single-frame intensity images [14,15,16]. In a study conducted in [17], wavefront sensing models were built using both a Multi-layer Perceptron (MLP) and CNNs with varying layers. The input to these models was a single-frame Point Spread Function (PSF), and the output was 2–400 orders of Zernike coefficients. All models, except for the MLP, achieved impressive results, underscoring the effectiveness of CNNs in addressing such problems. The accuracy of aberration prediction using single-frame images can rival that of dual-frame methods and holds promising prospects for practical optical systems due to its lower complexity.

In this paper, we establish an atmospheric turbulent laser transmission model to create a theoretical dataset. We propose a scheme that utilizes deep CNNs to map single-frame intensity images to 67-order Zernike coefficients. We compare the performance of three networks: ResNet [18], Xception [19], and ResNeXt [20]. Additionally, we study the effects of incorporating different attention mechanisms into the baseline network. Finally, we determine that the ResNeXt50 architecture, combined with the NAM (Normalization-based Attention Module) [21], is the optimal choice for constructing an atmospheric turbulence wavefront sensor for aberration detection. To validate the effectiveness and accuracy of this method in practical systems, we establish an indoor experimental system. Real-time aberration detection and correction based on the DLWS are performed, achieving a correction effect that closely approaches that of an AO system based on SHWS. Subsequently, we build an outdoor laser atmospheric transmission experimental platform and directly apply the indoor trained DLWS to the outdoor platform. The results demonstrate the practicality of the method presented in this article, as it can be easily migrated to different platforms.

## 2. Deep Learning Wavefront Sensing

### 2.1. DLWS

**Figure 1:**
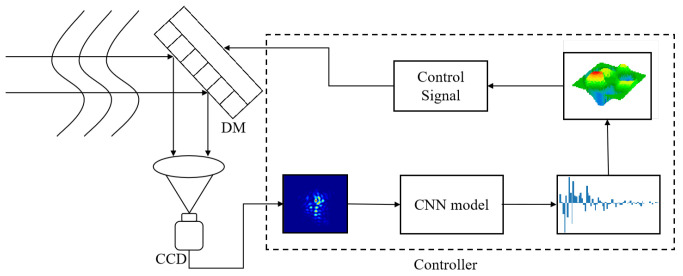
Workflow of an AO system based on DLWS.

Traditional AO systems primarily rely on SHWS for wavefront aberration detection. In contrast, deep learning-based wavefront sensing approaches require only a camera and a processor to achieve wavefront detection. This significant simplification of the optical path complexity and reduction in system costs make it applicable even in scenarios with weak or unevenly distributed beacon light intensities. Figure 1 illustrates a schematic diagram of an AO system incorporating DLWS. The system initially captures images using a CCD camera, which are then processed by the controller. By employing a Convolutional Neural Network (CNN), the controller conducts inference to obtain wavefront aberration coefficients. Subsequently, these coefficients are combined with a transformation matrix to generate control signals for implementing wavefront correction.

### 2.2. Network Research of DLWS

DLWS can be considered as a regression task, where images are mapped to coefficient vectors. Similar to image classification tasks, feature extraction from images plays a crucial role in this process. Deep learning, especially that of CNNs, has shown remarkable performance in general image processing tasks. The introduction of ResNet has made it possible to design deeper network structures, which has led to the development of excellent deep CNN for image feature extraction in various fields, including DLWS. Examples include [14] using Inceptionv3 and [15,22] using Xception to construct DLWS. In practice, the depth of a neural network is a critical factor for its performance. Deeper networks have the potential to extract more complex features, leading to better results. However, increasing network depth can sometimes saturate accuracy or even reduce it. ResNet addresses this challenge by introducing shortcut connections, which allow deep networks maintainance of identity mapping and prevention of performance degradation with increasing depth. On the other hand, Inception [23] optimizes network width by decomposing large convolutions into smaller ones, reducing computational complexity while enhancing representational power. Xception builds upon the Inception module by increasing the number of branches with 3 × 3 convolutions and adding residual connections. ResNeXt, inspired by ResNet, introduces group convolutions, combining residual structures with grouped convolutions to further improve network performance.

Attention mechanisms mimic human observation by enabling models to assign varying weights to different input elements, focusing on crucial information. This enhances model accuracy without significantly increasing computational and storage demands. In CNNs, attention mechanisms fall into spatial, channel, and mixed domains. A commonly used mechanism is a CBAM (Convolutional Block Attention Module) [24], which builds the Channel Attention Mechanism by adding parallel max-pooling layers based on SENet (Squeeze-and-Excitation Networks) [25]. It also applies attention to input features in both channel and spatial dimensions through the SAM (Spatial Attention Mechanism) [21], composed of pooling and convolutional layers. NAM follows a similar integration approach to CBAM but uses Batch Normalization scaling factors to represent weight importance, eliminating the need for fully connected layers and reducing computational costs. This paper also compares other lightweight attention mechanisms, such as SGE [26] and ECA [27,28,29,30].

In our study, we build DLWS models based on ResNet, Xception, and ResNeXt using a consistent dataset. With a 256 × 256 PSF frame as input and a set of 67-order Zernike coefficients as output, we compare the performance of the three models. The most effective network is then chosen as the baseline to analyze the impact of incorporating various attention mechanisms.

## 3. Numerical Simulation

### 3.1. Data Generating and Preprocessing

Atmospheric turbulence wavefront phase can be represented by a phase screen. In this paper, the spectral inversion method [31] is used to simulate the production of atmospheric turbulence phase screen. The basic principle is as follows: first, a complex Gaussian random number matrix is filtered by the square root of the atmospheric turbulence power spectrum, and then random phase of atmospheric disturbance can be obtained by performing Fourier transform on it [32,33]. The equation is as follows:

(1) $$\varphi \left(x,y\right)=C\underset{\infty }{\;\int \int \;}T\left(K_{x},K_{y}\right)\sqrt{\Phi (K_{x},K_{y})}e^{i\vec{r}\cdot \vec{K}}d\vec{K},$$

where *x*, *y* are the spatial coordinates, *C* is a constant, $\vec{K}$ is a wave vector, $\vec{K_{x}}$ and $\vec{K_{y}}$ are the components in the *x* and *y* directions, K=2π/λ, λ is the wavelength, *i* is an imaginary number, $\vec{r}$ is the space coordinate vector, $\left|\vec{r}\right|=\sqrt{x^{2}+y^{2}}$, $\Phi (K_{x},K_{y})$ is the atmospheric turbulence power spectrum, $T(K_{x},K_{y})$ is the matrix of complex Gaussian random numbers, φ(x,y) is the atmospheric turbulence wavefront aberrations.

The atmospheric turbulent distortion wavefront generated by the simulation can be expanded by a series of annular Zernike models [34].

(2) $$\varphi \left(x,y\right)=\sum_{i=1}^{l}a_{i}Z_{i}^{\varepsilon }\left(x,y\right),$$

where *l* is the model number, $a_{i}$ is the undetermined model coefficients, ε is the obscuration ratio, $Z_{i}^{\varepsilon }\left(x,y\right)$ is the Zernike model item in the annular domain.

**Figure 2:**
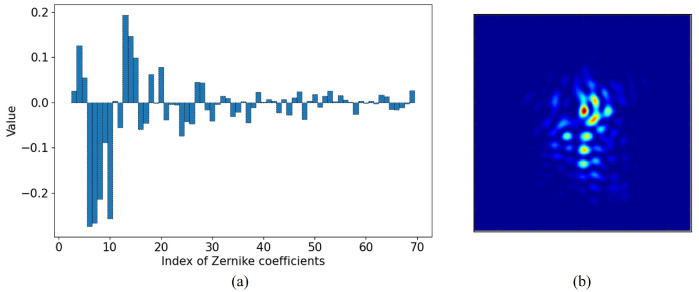
Example of sample pairs generated by simulation, (**a**) Zernike coefficients of wavefront aberration decomposition, (**b**) the far-field intensity image.

The number of selected items is *l*, and the mode decomposition of the given wavefront can be performed to obtain the mode coefficients.

(3) $$A=Z^{+}\varphi ,$$

where *A* is the model coefficient matrix, $Z^{+}$ is the inverse matrix of the Zernike model matrix in the annular domain. Considering that our wavefront corrector performs well in correcting aberrations up to the 70th order of Zernike coefficients, and based on the findings in reference [35], which indicate that neural networks can predict around 65 Zernike coefficients with high wavefront reconstruction accuracy, we establish the prediction of 67 Zernike coefficients in this paper. A set of 4–70 radial Zernike mode bases, denoted as $Z_{i}^{\varepsilon }\left(x,y\right)$, is selected. Mode decomposition is performed on the simulated circular random atmospheric turbulence wavefront phase, resulting in 67 mode coefficients as shown in Figure 2a. For DLWS in this paper, the input is assumed to be a PSF that has already been corrected for tilt; thus, tilt terms are not considered. In the case of theoretically generated datasets, images are cropped with the centroid position as the center. In practical experimental applications, a Fast Steering Mirror (FSM) is employed to eliminate tilt, causing the offset between the centroid of the far-field spot and the image center to approach zero.

According to the lens diffraction principle, the complex beam amplitude distribution on the focal plane of the lens is the Fourier transform of the distorted wavefront. Considering the influence of the lens aperture, assuming a uniform distribution of beam intensity on the focal plane, the complex beam amplitude can be calculated using the following equation [36]:

(4) $$E=FT(P\times e^{j\varphi }),$$

where *E* is the complex amplitude distribution on the focal plane of the lens, FT is the Fourier transform, and *P* represents the lens aperture function, which can be expressed as follows:

(5) $$P=\left\{\begin{array}{cc} 1, & r\times l_{0}<l\leq l_{0}\\ 0, & \;\mathrm{else}\; \end{array}\right.,$$

where *l* is the distance from any point on the pupil plane to the center, $l_{0}$ is the pupil size, and *r* is the central obscuration ratio. Therefore, the far-field diffraction image corresponding to the circular random atmospheric turbulence wavefront can be obtained, as shown in Figure 2b.

### 3.2. Training and Results

The paper conducted simulations with the atmospheric coherence length, r0, randomly set within the range of 5 cm to 15 cm, while the pupil diameter *D* remained consistent at 54 mm, matching the indoor experimental platform. The paper simulated a total of 120,000 data pairs with the aforementioned settings. The dataset was partitioned into training and validation sets in a 5:1 ratio. Additionally, 20,000 extra data pairs were simulated, and a random subset was selected for use as the test set. The training was performed on a computer equipped with an Intel Xeon 4214R CPU operating at 2.4 GHz, 256 GB of RAM, and four NVIDIA Tesla V100 GPUs with 128 GB of VRAM. Stochastic Gradient Descent (SGD) with a momentum value of 0.9 was used for optimization, facilitating faster convergence during training. A decay rate of $10^{-4}$ was applied to adaptively adjust the learning rate as training progressed, optimizing the model parameters. The initial learning rate was set at 0.1, dynamically decreasing during training to accommodate data characteristics and enhance the training process.

The chosen loss function for network training was the L1 (*MAE*) loss, also known as the mean absolute error. This loss function measures the absolute difference between predicted and target values, promoting robust training and minimizing the impact of outliers. The formulas for *MAE* and *MSE* are given by Equation (6), where *m* represents the number of samples, $pred_{i}$ represents the predicted value for the *i*th sample, and $gt_{i}$ represents the true value for the *i*th sample.

(6) $$\begin{array}{cc} MAE & =\frac{1}{m}\sum_{i=1}^{m}\left|{pred}_{i}-{gt}_{i}\right|,\\ MSE & =\frac{1}{m}\sum_{i=1}^{m}{\left({pred}_{i}-{gt}_{i}\right)}^{2}. \end{array}$$

**Figure 3:**
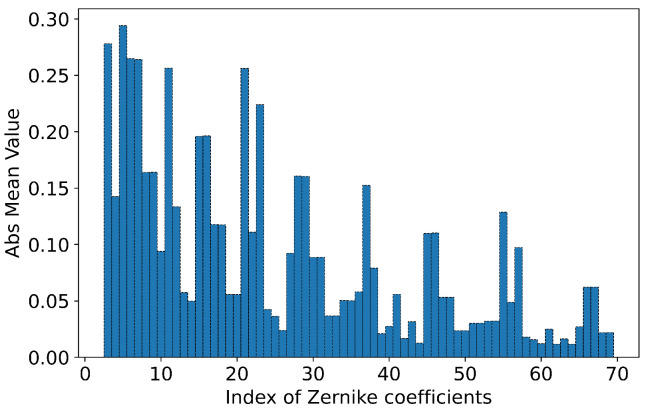
Average absolute value of each Zernike coefficient in the training set.

Compared to L2 (*MSE*), L1 regularization helps mitigate outlier influence. Although L1 regularization assigns the same gradient to errors of different magnitudes, potentially affecting late-stage convergence, we addressed this by automatically reducing the learning rate when errors were small. Before training, we conducted a statistical analysis of the average values of each coefficient in the training set, as illustrated in Figure 3.

It was observed that Zernike coefficients, exhibiting characteristics of atmospheric turbulence aberration, displayed significant variability. To ensure that the model assigns equal importance to each order, we normalized each coefficient by dividing it by the average value of its respective order. Additionally, input images were normalized to the range of 0–1.

**Figure 4:**
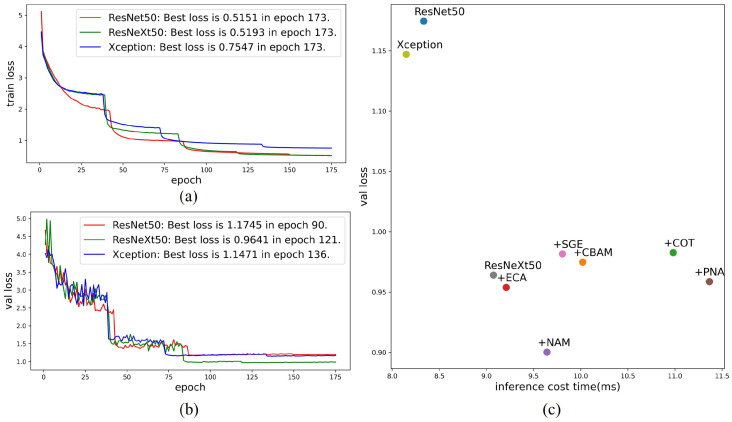
Figures depicting the training and validation loss of different backbone CNN networks, as well as performance comparison charts after incorporating different attention mechanisms. (**a**) Training loss of the three networks, (**b**) validation loss of the three backbone CNNs, (**c**) performance comparison after adding different attention mechanisms to ResNeXt50, where the x-axis represents inference latency and the y-axis represents validation error.

Figure 4a,b display the training and validation errors of the three models during training. These figures clearly indicate that all three models reach their optimal performance levels, with ResNeXt50 demonstrating the highest accuracy. As a result, ResNeXt50 was selected as the baseline network for further investigation into the impact of attention mechanisms on performance. Attention modules CBAM [24], COT (Contextual Transformer) [37], NAM [21], ECA [30], PNA [27], and SGE [26] were integrated into the baseline network and trained accordingly. Figure 4c illustrates the performance of these attention mechanisms on the validation set.

Notably, the incorporation of the NAM attention module into ResNeXt50 significantly improved wavefront aberration identification accuracy, with an insignificant increase in inference time (less than 1 ms). This indicates that the advantages of including the NAM attention module outweigh its impact on computational efficiency, making it a valuable addition to the ResNeXt50 model for wavefront sensing applications. We conducted tests on the network models using the test set, and Table 1 presents the average errors for each network on both the validation and test sets. It is evident that on the test set, all models achieved results similar to those on the validation set. The inclusion of NAM in ResNeXt50 delivered the best performance.

**Figure 5:**
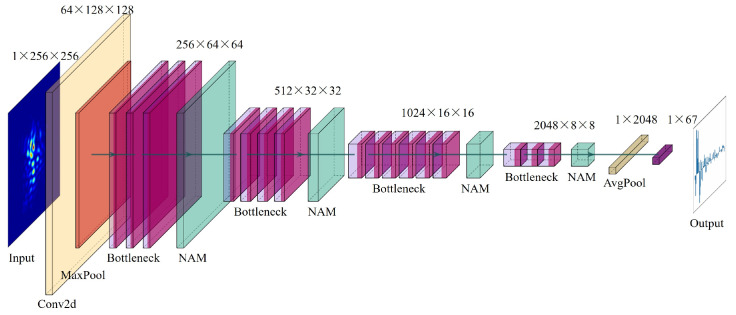
Network architecture diagram of ResNeXt-NAM.

Figure 5 provides a visual representation of the network architecture employed in the study. In this research, the NAM module was incorporated after each residual block, enabling attention mechanisms to be applied to feature maps at multiple scales. This utilization of multi-scale feature information proves to be highly effective in enhancing the accuracy of the model. Furthermore, to optimize the model for deployment, it was quantized and implemented using the TensorRT [38] tool. Remarkably, the quantized model achieved an impressive processing speed of 400 frames per second (FPS) on a computer with an Intel i7 10700 CPU running at 2.9 GHz, with 32 GB of RAM, and a NVIDIA RTX 2080TI GPU with 11 GB of VRAM. This demonstrates the model’s efficient and real-time performance in practical scenarios.

## 4. Atmospheric Turbulence Aberration Correction Experiment

### 4.1. Experimental Platform

**Figure 6:**
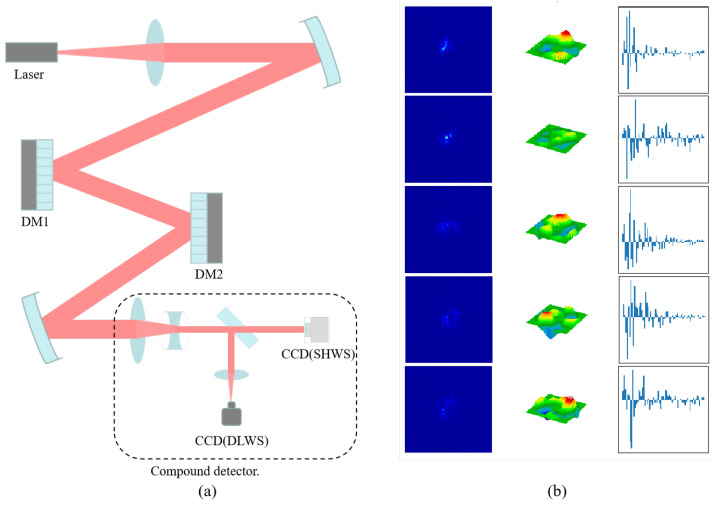
Indoor experimental platform and examples of the collected samples. (**a**) The indoor experimental platform. The composite detector is capable of simultaneously capturing far-field intensity images and wavefronts. (**b**) Examples of samples collected by the platform. From top to bottom, three randomly visualized sample sets; from left to right, PSF of sample pairs, corresponding wavefront, and decomposed Zernike coefficients.

To validate the effectiveness of the proposed ResNeXt-NAM wavefront sensor, we established two experimental platforms. Figure 6a illustrates the indoor laser transmission experimental setup, comprising a laser, a beam expander, Deformable Mirror (DM), compound detector (consisting of a SHWS and a far-field detector for simultaneous capture of wavefront and far-field intensity images), and a mirror. The number of subapertures for the Shack–Hartmann Wavefront Sensor (SHWS) is 36 × 36, with each subaperture having a resolution of 64 × 64, and the frame rate is 120 FPS. This platform serves for sample collection and model verification purposes. During sample collection, DM1 simulates atmospheric turbulence aberrations, and the compound detector records both far-field and wavefront images, with the wavefront decomposed into 67 Zernike coefficients. The collected sample library is showcased in Figure 6b. For verification, DM1 continues to simulate atmospheric turbulence aberrations, while DM2 performs real-time aberration correction. The correction signal can be derived from either the SHWS or the DLWS.

**Figure 7:**
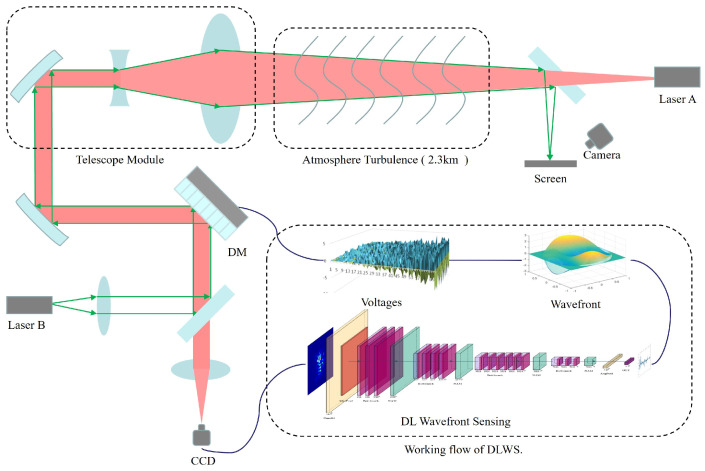
Schematic diagram of the experimental platform for atmospheric turbulence correction based on DLWS.

The outdoor laser transmission experimental platform comprises two conjugate optical paths. The laser transmission path is situated at a height of no more than 100 m above the ground, with a transmission distance of 2.3 km. Laser A serves as the beacon light source, and the telescope system captures the beacon light, directing it to the CCD to obtain far-field intensity images. These images are then input into the ResNeXt-NAM wavefront sensor. After wavefront reconstruction, the generated voltage is applied to the wavefront controller for aberration correction. Laser B is transmitted through the system and reaches the imaging plate near Laser A. By observing the spot image on the plate, the system’s correction effect can be easily evaluated. The schematic diagram of the experimental platform is presented in Figure 7.

### 4.2. Experiment and Results

On the indoor experimental platform, a total of 240,000 samples were collected and divided into training, validation, and test sets in a ratio of 4:1:1. Notably, during training, the background image without light is subtracted from each PSF to mitigate the influence of detector noise. For this training, we utilized the model trained on the theoretical dataset as a pre-trained model.

**Figure 8:**
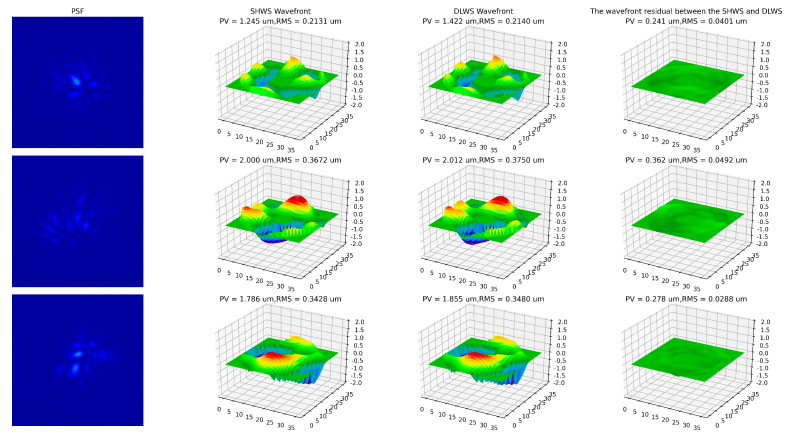
The comparison between the wavefronts obtained by SHWS and DLWS. From **left** to **right**, it consists of the PSF, the wavefront detected by SHWS, the wavefront detected by DLWS, and the residual between the two wavefronts. From **top** to **bottom**, the results collected at three different moments in time.

This approach significantly improved convergence efficiency and reduced training time. Upon completing the training, the model achieved a minimum error of 0.1023 on the validation set. The best-performing model demonstrated a test error of 0.1027, which closely matches its performance on the validation set. Figure 8 provides a comparison of the simultaneous wavefront detection results obtained by SHWS and DLWS at three different time points.

**Figure 9:**
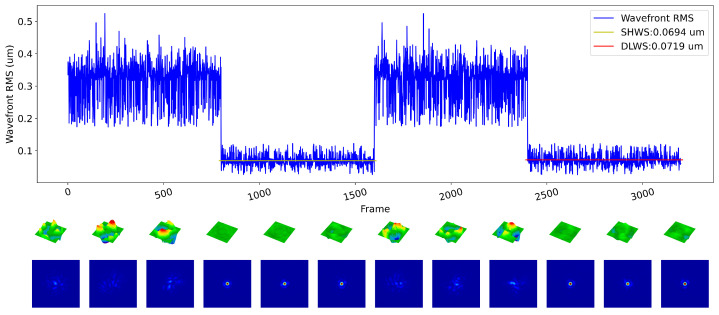
Comparison and visualization of different methods in open-loop and closed-loop configurations on the indoor experimental platform. The line graph shows the wavefront RMS values for four stages: open loop, closed loop based on SHWS, open loop, and closed loop based on DLWS. Below, visualizations are presented for randomly selected wavefronts and far-field intensity images corresponding to each stage.

Real-time turbulent aberrations were generated using the DM, and wavefront sensing and closed-loop correction were performed using both the SHWS and the DLWS. Figure 9 presents a comparison of the closed-loop results. After closed-loop correction based on the SHWS, the average wavefront RMS was measured to be 0.0694 um. In contrast, the average wavefront RMS after closed-loop correction based on the DLWS was 0.0719 um. These results indicate that our method achieved a level of closed-loop performance comparable to that of the SHWS in the system.

**Figure 10:**
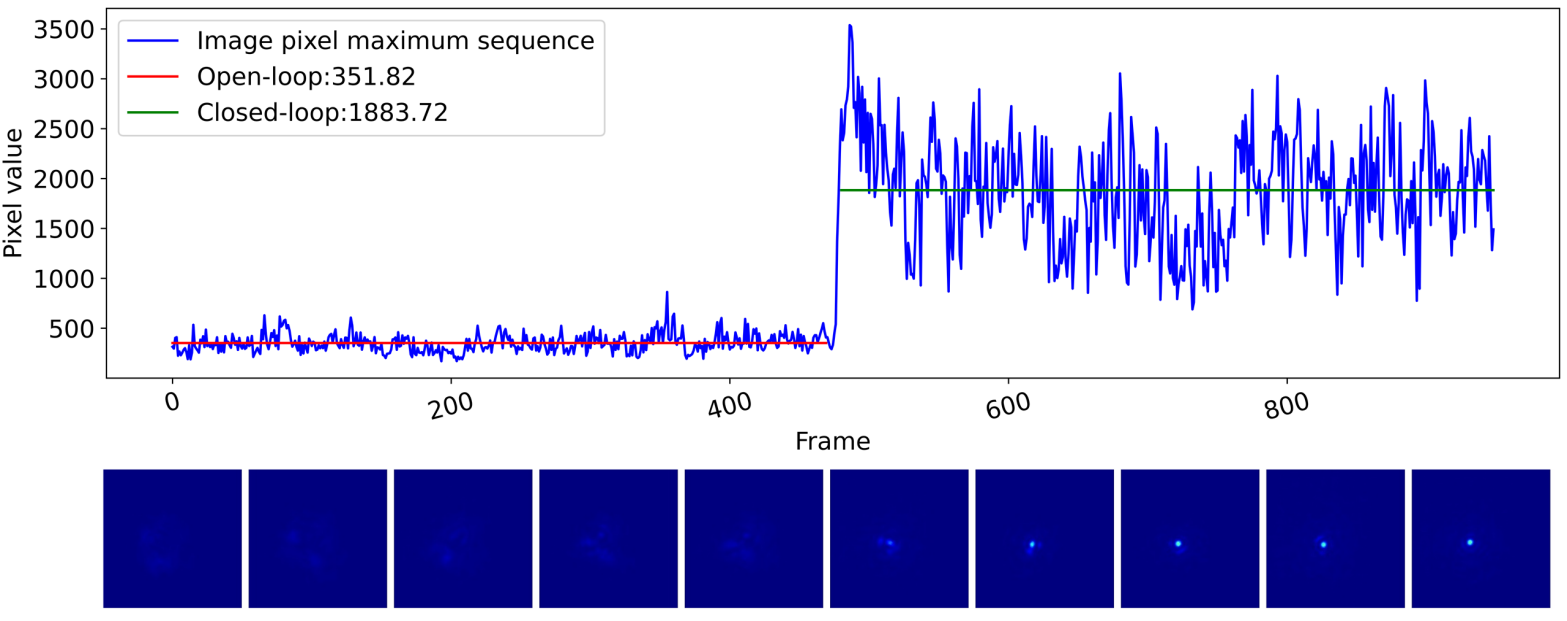
Comparison of open-loop and closed-loop experiments for atmospheric turbulence correction based on DLWS. The curve shows the peak statistics of the intensity images of laser B at the target point during the open-loop and closed-loop experiments. Below are randomly selected intensity images corresponding to each state.

During the design of the experimental platforms, we deliberately ensured that the system parameters were similar for both platforms. The telescope system on the outdoor platform has a magnification of 6 and an aperture of 360 mm. To ensure it had the same entrance pupil and focal length as the indoor platform detection system, we used the same detection system and added a 54 mm aperture. Consequently, we directly applied the well-established ResNeXt-NAM wavefront sensor, developed and validated on the indoor platform, to the outdoor platform for detecting atmospheric turbulence. The obtained wavefront was then utilized to correct the aberrations in the transmission optical path. Subsequently, we captured images on the imaging plate at the target point and selected a segment for statistical analysis of the peak intensity. Figure 10 illustrates experimental results over a period with an average $r_{0}$ of 10.2 cm ($D/r_{0}=3.53$). The maximum pixel values of the PSF detected at the target point during this period were recorded.

In the open-loop configuration, the average peak intensity was merely 351.82, resulting in a scattered and blurred light pattern in the corresponding image. However, in the closed-loop configuration, the average peak intensity significantly increased to 1883.72, allowing for a clear and well-defined pattern resembling an Airy disk in the image. These findings strongly indicate the effective correction of atmospheric turbulence by our system, leading to a remarkable enhancement in beam quality. It is noteworthy that the DLWS employed in this experiment was transferred from the indoor platform without the need for additional data acquisition or training, highlighting the remarkable generalization capability of our proposed method.

## 5. Conclusions

In this article, we introduced a novel deep learning wavefront sensing model known as ResNeXt-NAM. This model was developed through extensive comparative experiments involving different network backbones and attention mechanisms to achieve optimal results. The method not only excels when applied to the theoretical atmospheric turbulence dataset established in this study, but also demonstrates performance on par with that of SHWS when tested on experimentally collected data. By transplanting the deep learning sensor trained in the laboratory to a kilometer-level experimental platform, we conducted wavefront detection using beacon light intensity images to compensate for aberrations in the transmitted light. The results obtained at the target point indicate that the closed-loop correction method has a significant suppressive effect on atmospheric turbulence under experimental conditions, with the average peak intensity of the spot increasing by 5.35-fold post correction.

These findings highlight the effectiveness of the DLWS proposed in this article for correcting atmospheric turbulence. We anticipate that further improvements can be achieved by collecting additional samples on the outdoor platform and incorporating them into the original dataset. However, acquiring real-world data under practical application scenarios can be challenging. Therefore, the development of a dedicated indoor platform for DLWS training may be a viable approach.

## Figures and Tables

**Table 1:** The performance of different networks on the test set corresponding to the model with the best loss on the validation set. FPS (Frames Per Second) reflects the inference speed of the model in predicting the time spent on a single frame.

Models	ResNet50	Xception	ResNeXt50						
			\	+CBAM	+COT	+ECA	+PNA	+SGE	+NAM
Val Loss	1.1745	1.1471	0.9641	0.9748	0.9826	0.9539	0.9587	0.9816	**0.9002**
Test Loss	1.2262	1.1996	0.9702	0.9803	1.012	0.9592	0.9601	0.9871	**0.9062**
FPS	121.4	**124.3**	111.4	100.8	91.9	109.8	89.2	102.1	104.8
Train time (epoch/s)	516	**432**	621	668	762	661	723	662	692

## Data Availability

Data underlying the results presented in this paper are not publicly available at this time but may be obtained from the authors upon reasonable request.
